# Development of an interactive dashboard for gun violence pattern analysis and intervention design at the local level

**DOI:** 10.1093/jamiaopen/ooad105

**Published:** 2023-12-11

**Authors:** Rashaud Senior, Lisa Pickett, Andrew Stirling, Shwetha Dash, Patti Gorgone, Georgina Durst, Debra Jones, Richard Shannon, Nrupen A Bhavsar, Armando Bedoya

**Affiliations:** Duke University Health System, Durham, NC 27710, United States; Duke Health Technology Services, Durham, NC 27710, United States; Duke University Health System, Durham, NC 27710, United States; Duke University Health System, Durham, NC 27710, United States; Duke Health Technology Services, Durham, NC 27710, United States; Duke University Health System, Durham, NC 27710, United States; Duke Health Technology Services, Durham, NC 27710, United States; Duke University Health System, Durham, NC 27710, United States; Duke Health Technology Services, Durham, NC 27710, United States; Duke University Health System, Durham, NC 27710, United States; Duke University Health System, Durham, NC 27710, United States; Duke University Health System, Durham, NC 27710, United States; Department of Medicine, Duke University Hospital, Durham, NC, United States; Duke University Health System, Durham, NC 27710, United States; Duke Health Technology Services, Durham, NC 27710, United States

**Keywords:** gun violence, social determinants of health, electronic health record, dashboard analysis

## Abstract

**Introduction:**

Gun violence remains a concerning and persistent issue in our country. Novel dashboards may integrate and summarize important clinical and non-clinical data that can inform targeted interventions to address the underlying causes of gun violence.

**Methods:**

Data from various clinical and non-clinical sources were sourced, cleaned, and integrated into a customizable dashboard that summarizes and provides insight into the underlying factors that impact local gun violence episodes.

**Results:**

The dashboards contained data from 7786 encounters and 1152 distinct patients from our Emergency Department’s Trauma Registry with various patterns noted by the team. A multidisciplinary executive team, including subject matter experts in community-based interventions, epidemiology, and social sciences, was formed to design targeted interventions based on these observations.

**Conclusion:**

Targeted interventions to reduce gun violence require a multimodal data sourcing and standardization approach, the inclusion of neighborhood-level data, and a dedicated multidisciplinary team to act on the generated insights.

## Introduction

Gun violence is an ongoing issue in the United States, causing over 40 000 deaths and 76 000 injuries annually,^1^ disproportionately in socio-economically disadvantaged areas.^2^ The total number of gun-related deaths in the United States rose to a record high in 2020, accounting for a 14% increase from the prior year, a 25% increase over the prior 5 years, and a 43% increase over prior the last decade.^3^ A study from 2021 using community- and state-level data strongly suggests the importance of neighborhood-level analysis of this issue, finding that gun violence was “higher in counties with both high median income and higher levels of poverty; [whereas] poverty did not seem related to gun violence rates in counties with relatively low median incomes.”^4^ Similarly, another study found that Black residents experienced higher rates of firearm-related homicides even when the relative deprivation index was the same, suggesting a relationship with interconnected social issues such as poorer institutional resources and disparate law enforcement responses in Black neighborhoods in particular.^2^ The combination of data around the social determinants of health (SDOH) and clinical outcomes better helps us understand the underlying issues related to gun violence.

Health systems, generally struggle to innovate^5^ and have not incorporated SDOH data into their analyses despite the many benefits of better digitization and an expanded understanding of social factors’ contributions to health outcomes.^6–8^ Fortunately, informatics-based tools can help automate the inclusion of various data^9^ despite barriers within the implementation lifecycle.^10–14^ In this context, gun violence might be best addressed through understanding SDOH-related patient factors and partnering with community stakeholders equipped to address these social needs.^15–17^

At Duke University Health System (DUHS), a working group was formed within the Collaborative to Advance Clinical Health Equity (CACHE) program to address this issue. They sought to define the population at risk for gun violence and develop a data-driven, informatics-based approach to inform interventions in Durham, NC, United States.

## Methods

The goal of this CACHE program is to identify clinical, individual, and neighborhood contextual and built environment factors that may increase an individual’s risk to become a victim of gun violence in Durham, NC, United States. To that end, the primary goal was the development of an informatics process to source data and communicate this information to various stakeholders.

### Gun violence data

Victims of gun violence were identified from the Duke Trauma Registry with a chief complaint of gunshot wound (GSW) with penetrating injury type. ICD-10 external codes (Table S1) were included for all penetrating GSW types that were not related to suicide. A SQL-based query was used to identify these patients within background Clarity tables of the Epic electronic health record (EHR) instance at DUHS to generate a formal patient list with basic demographic information (Table 1). Additional gun violence data came from the Durham Police Department (DPD), who compiled a set of service calls data, including date, location, nature of the call, and disposition, from 2018 through September 2021 using publicly available data.^18^

**Table 1.** Baseline population demographics.

Total number of encounters	7786
Total number unique patients	1169
Age	
<18	159 (13.6%)
18-34	688 (58.7%)
>34	326 (27.8%)
Sex	
Male	992 (84.9%)
Female	177 (15.1%)
Race	
Caucasian/White	152 (12.9%)
Black/African-American	910 (77.5%)
Asian & Pacific Islander	2 (0.2%)
Native American/Alaskan	4 (0.3%)
Multiple Races	10 (0.9%)
Not reported/declined	43 (3.7%)
Unavailable/null	3 (0.3%)
Other	50 (4.3%)

### Clinical data

After identifying the patient cohort, we pulled various types of clinical data across EHR tables as the project was discussed with executive leadership. This included demographic, diagnoses, medication, and healthcare utilization data. Successive iterations of the dashboards, informed by these discussions, included additional data such as mental health diagnoses, active and prior medication sub-classes, medication prescription sources, the frequency of visits with primary care and behavioral health providers, frequency of Emergency Department and Urgent Care visits, and recent pain medication prescription dosing and frequency.

### Non-clinical and SDOH data

We linked clinical data with extant non-clinical data obtained from multiple sources. The list of local businesses, schools, churches, and their locations was obtained from ReferenceUSA.^19^ Area level deprivation data were comprised of the Area Deprivation Index (ADI)^20^^,^^21^ and the Social Vulnerability Index (SVI),^22^ both using 2018 data. Data from the Elixhauser Comorbidity Index for fiscal year 2020^23^ were also incorporated. Additional SDOH data were downloaded from Duke’s Clinical & Translational Science Institute’s Social, Environmental, and Equity Drivers (SEED) Health Atlas initiative,^24^ which was developed from prior work done within Duke to compile non-clinical data for research purposes. Socioeconomic data for Durham County were obtained from the Durham Open Data Portal.^25^ Census block group data were obtained from the US Census Bureau.^26^

### Data linkage and dashboard creation

**Figure 1.**
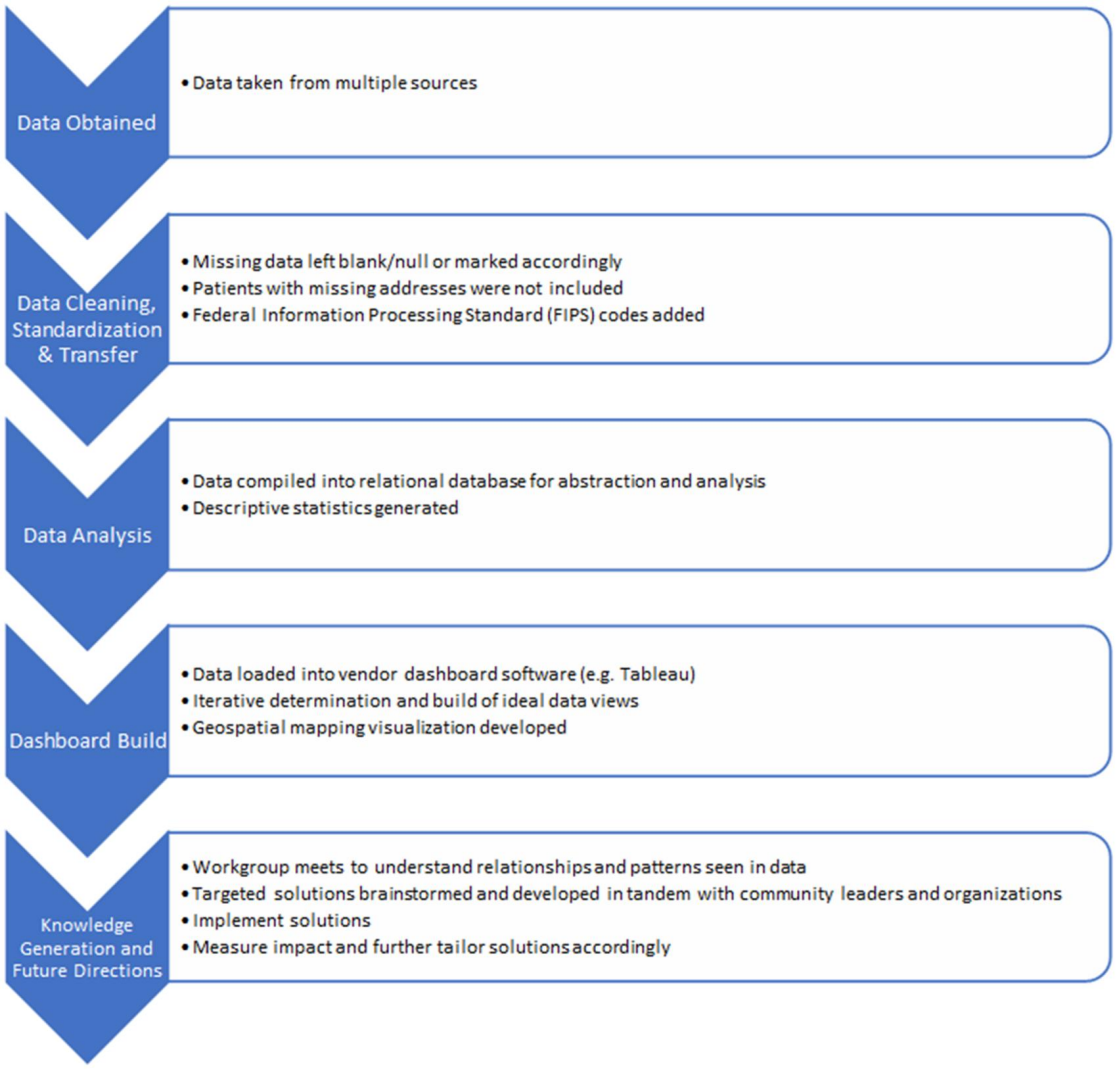
Data pull and transformation process.

Data were then cleaned and standardized. All data were required to have Federal Information Processing Standard (FIPS) codes as part of their respective datasets to support linkage and geospatial mapping at a common census area level. Patient-level data were linked across datasets using a combination of available demographic information. Patients for whom we did not have an address were excluded from the dashboards as were fatalities and self-inflicted GSWs. Table 2 provides a summary of our data sources. Missing data were left as blank/null values in their respective datasets and summary dashboards to prompt discussion to address them. For example, missing values in the ADI marked as “Unknown or Outside NC” led to discussions with other analytics groups within Duke, a new vendor relationship, and more complete patient geocodes. Each dataset was then compiled into a relational database architecture for abstraction (Figure 1).

**Table 2.** Data sources.

	Category	Source	Details
Gun violence	Base Patient List	Duke Internal Trauma Registry	Used specific keywords within ICD-10 codes for ED encounters
	Local Crime Data	Durham Police Dept	Service calls, location, disposition, etc., obtained from DPD website
Clinical data	Diagnoses (active and prior)	Local Electronic Health Record	
	Medications (active and prior)	Local Electronic Health Record	Includes subclasses as determined by clinicians on dashboard development team
	Prior healthcare visits	Local Electronic Health Record	
Non-clinical and SDOH data	Local businesses, schools, churches	ReferenceUSA	
	Area Deprivation Index	University of Wisconsin School of Medicine and Public Health	
	Social Vulnerability Index	Centers for Disease Control and Prevention	
	Elixhauser Comorbidity Index	Agency for Healthcare Research and Quality	
	Miscellaneous SDOH data	Duke’s Clinical & Translational Science Institute’s Social, Environmental, and Equity Drivers (SEED) Health Atlas	Developed internally but published on public website
	County-level data	County public data portal	
	Census Block Groups	US Census Bureau	

**Figure 2.**
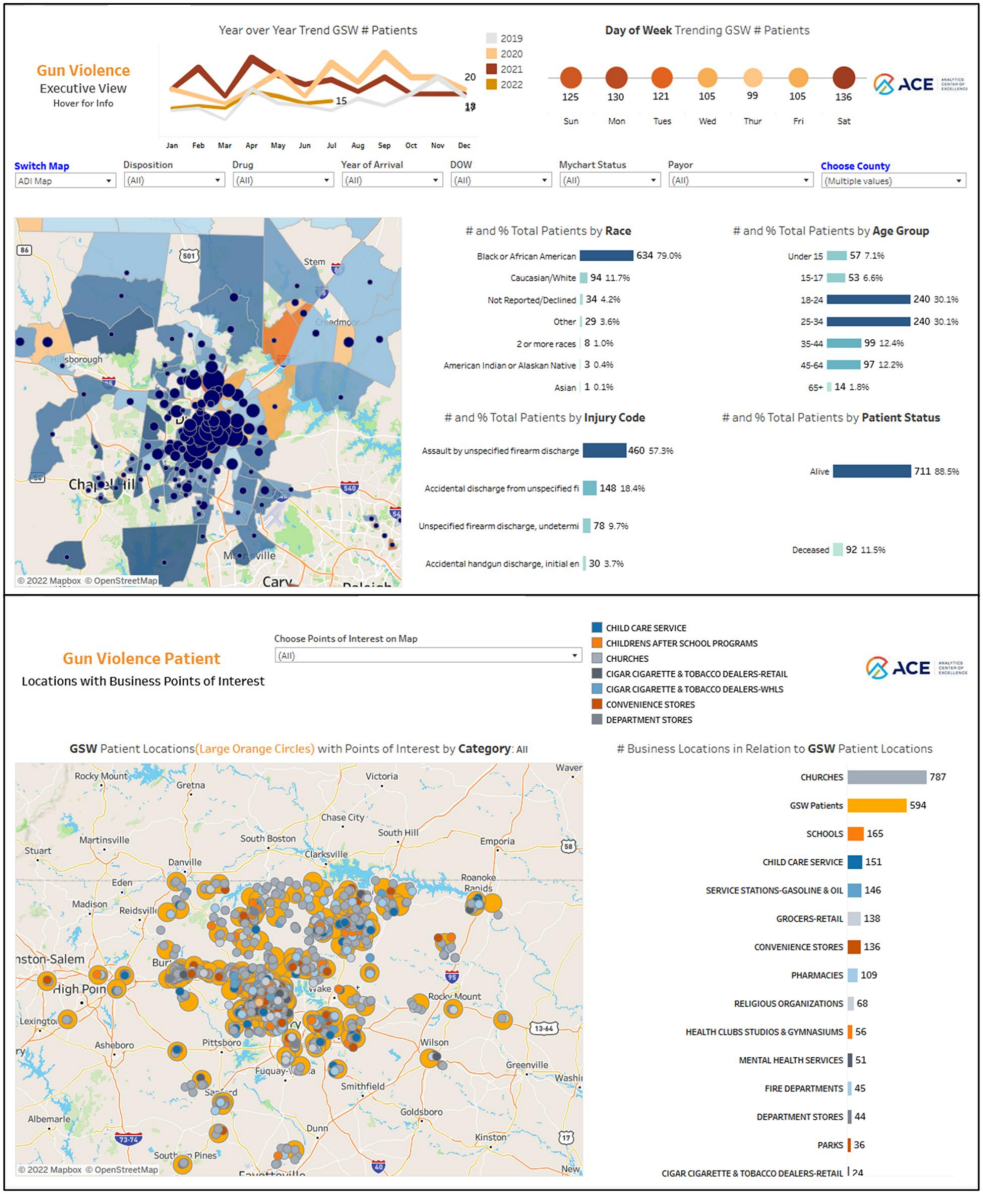
Dashboard screenshots.

A customized set of dashboards was then built to analyze, report, and visualize the data using Tableau software (version 2021.4). Descriptive statistics were generated as appropriate. The dashboards were built to facilitate sub-population identification through data filters and sorting performed by the end-user (Figure 2). Using natively integrated Mapbox OpenStreetMap as well as Google Maps functionality, they include a geospatial mapping visualization that combines and overlays contextual data such as patient location density, local business types and schools, ADI and SVI scores, and police service call locations. The dashboards underwent several rounds of review leading to further customizations in their structures, functions, and underlying data.

## Results

The dashboards contained data on 1152 distinct patients from the Trauma Registry between September 2019 and July 2022, with data from 7786 encounters going back to January 2016. They were predominantly aged 18-34 (60%), male (85%), and Black/African-American (78%) as summarized in Table 1 (variations attributed to updated demographic data since initial analysis). For those with multiple GSW events, the age at the first event was used in analysis. Select clinical variables were included in the dashboard using the initial GSW presentation to DUHS as the “index event” (Figure 2).

We were able to identify some clinical factors common in victims of gun violence based on clinical experience and prior literature. For example, at the patient level, prior to the index event, 403 (35%) distinct patients were prescribed an opioid, benzodiazepine, or psychiatric medication; of those, 145 (36%) were on a psychiatric medication and 357 (88.6%) were on an opioid or benzodiazepine. At the encounter level, for identified departments, these patients missed 30% of 560 total primary care encounters and 16% of 64 psychiatric/behavioral health encounters prior to the index event.

**Figure 3.**
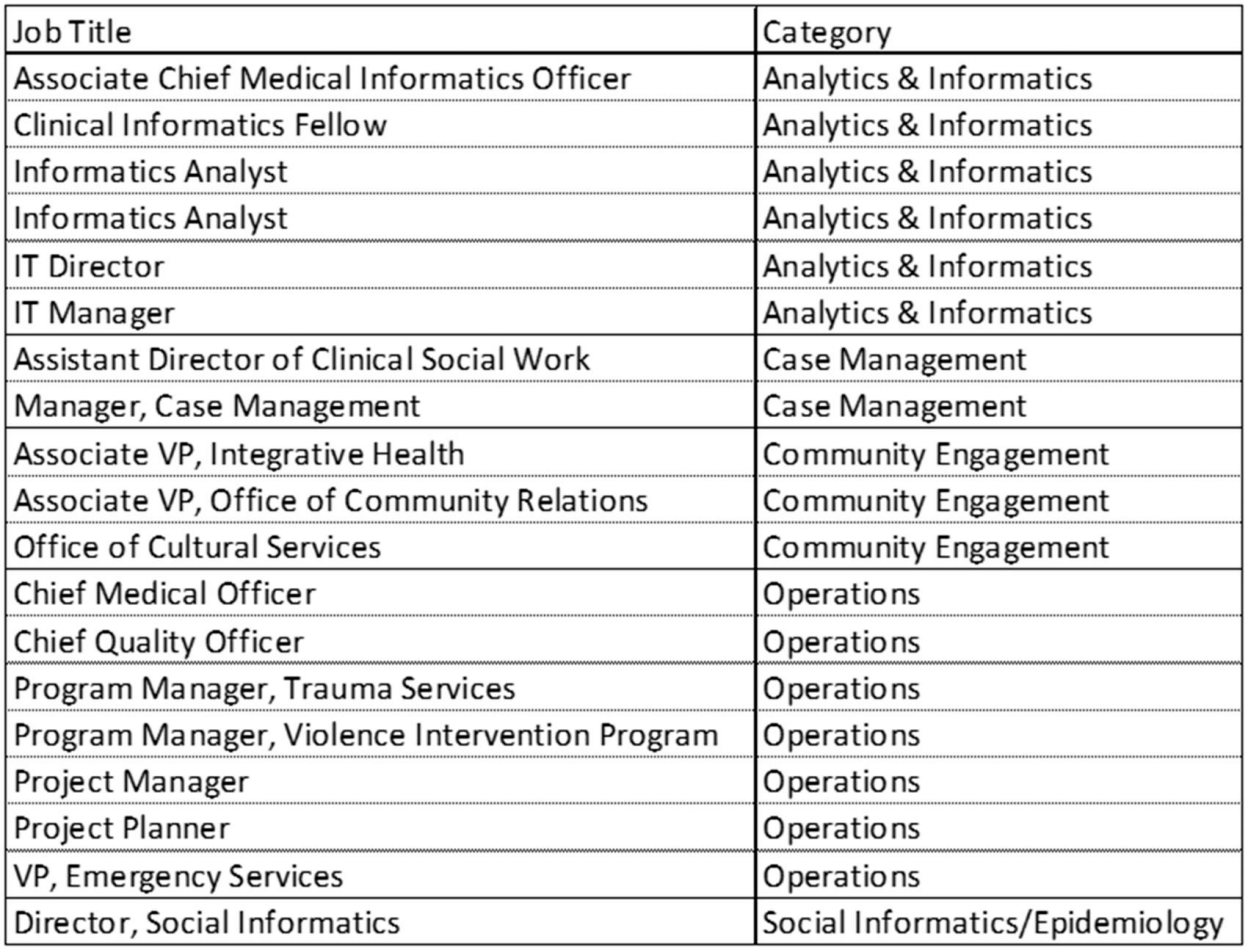
List of executive team member titles and categories.

To help guide decision-making and resource allocation at DUHS, a small multidisciplinary team was formed to review the dashboards. The initial group included individuals from different areas within the institution at various levels of management and, over time, involved additional experts and community leaders (Figure 3). Importantly, the team included subject matter experts in community-based interventions, epidemiology, and social sciences—their inclusion is particularly novel compared to traditional quality improvement initiatives, which are often based solely on clinicians as the subject matter experts.

### Sensitivity analysis

We compared baseline characteristics of individuals who are homeless and found that they were similar to the overall population. Of the 123 individuals excluded due to missing addresses, they were predominantly aged 18-34 (61%), male (92%), and Black/African-American (78%), while 25% of this population were deceased per the most recent analysis.

## Discussion

Our work highlights the utility and importance of combining various data types into customizable dashboard software to address social issues. Viewing such issues objectively by understanding the affected population through data analysis and trending allows those with the resources to identify and implement tailored interventions to maximize long-term impact. To date, we have found few other informatics tools that provide these data on this type of platform.

Our tool allows users to review various clinical and non-clinical data simultaneously and to trend them over time. Importantly, customizable visualizations help them make connections between the disparate data sources. For example, knowing the location of various businesses (eg, liquor stores, convenience stores), churches, and schools, as well as supplemental data (eg, internet connectivity, unemployment, housing or car ownership, or environmental exposures) provides opportunities to understand the affected population more deeply. Our incorporation of crime type and location data is especially notable for its potential to inform public interventions, as evidenced by recent work done by the Minnesota Department of Health to develop dashboards similarly merging these data types.^27^

Using these different views, the executive team gleaned actionable insights from the data, allowing for customized interventions at the institutional level. Accordingly, they have generated a list of recommendations for the institution to act upon, including regular community needs assessments, tracking gun violence event rates, and monitoring changes in social conditions at the census-tract level (eg, educational attainment, number of high-risk youth receiving intervention services, number of individuals receiving living-wage employment, etc.).

Aside from providing recommendations, the executive team has planned several direct community-level interventions. The Hospital-based Violence Intervention Program is a grant-funded intensive case management intervention providing wraparound services for victims of gun violence and their immediate family/friends. Led by the Office of Community Health, the team will meet with public and private groups to help centralize and coordinate gun violence interventions in Durham. To help combat a lack of educational and career growth opportunities that may lead to gang membership, the team plans to help enroll a small cohort of at-risk community members in GED classes, while offering after-school programming for school-age children.

The EHR alone is insufficient for this type of work. While it contains much of the data needed, that data are not standardized, nor is it consistently collected and implemented in clinical workflows. At our institution, for example, despite having an SDOH module within our EHR, it is rarely used by clinicians. Further, individual-level factors may not be enough to determine the root issues resulting in gunshot incidents; neighborhood- and community-level data are crucial to the full picture. A summary of lessons learned is as follows:

- The EHR alone is insufficient; neighborhood-level contextual data are needed.
- Data heterogeneity (ie, non-standardization) must be addressed and reconciled as data are taken and combined from multiple sources.
- A multidisciplinary team is needed for targeted interventions—it should include various clinical and non-clinical subject matter experts and executive leadership at the institution and community levels.
- Data presentation/visualization is key to gaining actionable insights.

Of note, this work has several limitations. Identifying the cohort of gun violence victims retrospectively does not directly predict those at risk of future gun violence. Therefore, it is likely that some at-risk sub-populations are missed in these analyses. Further, this work does not capture those in the community with “near miss” gun violence events or injuries for which people did not present to the emergency department. We also miss those who died prior to presentation to the hospital as well as the person who pulled the trigger. Fatalities were excluded from the initial dashboards as one goal for this project was to prevent a recurrent incident, requiring interviews with victims, and for ongoing assessments of intervention efficacy based on future data collections only possible in living patients. As the focus on this project was on gun violence homicides, suicide victims were excluded as they are fundamentally different in many aspects to homicide victims. Our approach is amenable to incorporating fatality and suicide data in future iterations of the dashboards, particularly as we build new key partnerships with relevant entities (eg, the state’s Medical Examiner’s Office) and develop existing ones (eg, obtaining additional data from the Police Department).

The lack of consistent patient-level SDOH data that would otherwise provide a picture of patient’s social background (eg, housing status, educational attainment, employment, etc.) hampers the ability to develop targeted interventions. The team excluded patients without addresses to maximize dataset linkages and insight generation, acknowledging that this population is an important one requiring a more nuanced approach to develop effective interventions. Additionally, the insights gleaned from the data (and, therefore, resulting actions) are wholly dependent on the questions being asked of the data and the ability of those using the dashboards to answer them. Further, self-reported race/ethnicity data and gender identity are inconsistently captured during medical visits and is therefore incomplete. We also note that the generalizability of our work is limited as it is partially dependent on the generation of data sets unique to our institution and the years spent curating them for our local population. However, we emphasize that the focus here be placed on the overall approach to gathering and combining data from multiple sources to better understand the local gun violence population to better inform targeted interventions.

## Conclusion

The moratorium on gun violence research has been lifted and federal funding is now being directed to it^28^^,^^29^; we expect to see more data-centered analyses and approaches to the gun violence issue in our country. Both national and local efforts to monitor and intervene on these newly-acquired data may follow a similar process to that presented here with the intention to develop complete automation in a future state.

Next steps include automating the regular updating of the underlying data in the dashboards. Ideally, this would include higher-level leadership action to develop lasting partnerships with organizations from which we have sourced the data to maintain data flow. As the impact of this work is determined, quantified, and communicated with leadership, more internal resources might be used for further process automation and augmentation. Further work in this area also would involve interventions addressing the historical and structural causes of higher gun violence rates among Black men. While they are beyond the scope of this paper, other groups have studied them in detail and provide meaningful contributions to the literature.^30–32^

## Supplementary Material

ooad105_Supplementary_Data

## Data Availability

The data underlying this article from the DUHS EHR cannot be shared publicly due to privacy and HIPAA restrictions. Datasets sourced from the public domain include: Durham Police Department (https://www.durhamnc.gov/719/Crime-Statistics), ReferenceUSA (accessed via Duke Libraries at https://idn.duke.edu/ark:/87924/r3gb21j98), Area Deprivation Index (DOI 10.1056/NEJMp1802313 and https://www.neighborhoodatlas.medicine.wisc.edu/), Social Vulnerability Index (https://www.atsdr.cdc.gov/placeandhealth/svi/data_documentation_download.html), Elixhauser Comorbidity Index (www.hcup-us.ahrq.gov/toolssoftware/comorbidityicd10/comorbidity_icd10_archive.jsp), Duke’s Social, Environmental, and Equity Drivers (SEED) Health Atlas initiative (https://sdoh.duhs.duke.edu/), Durham Open Data Portal (https://live-durhamnc.opendata.arcgis.com/), and the US Census Bureau (https://catalog.data.gov/dataset/tiger-line-shapefile-2019-state-north-carolina-current-census-tract-state-based).
