# Supplementary material for: Development of an interactive dashboard for gun violence pattern analysis and intervention design at the local level
Source: JAMIA Open. 2023 Dec 11;6(4):ooad105. doi: 10.1093/jamiaopen/ooad105 (PMC10712903; doi:10.1093/jamiaopen/ooad105)
Supplement: ooad105_Supplementary_Data [file ooad105_supplementary_data.docx]

| **Intent** | **ICD 10 External Code** | **Description** |
| --- | --- | --- |
| Accidental | W32.0XXA | Accidental handgun discharge, initial encounter |
|  | W33.00XA | Accidental discharge of unspecified larger firearm |
|  | W33.01XA | Accidental discharge of shotgun, initial encounter |
|  | W33.02XA | Accidental discharge of hunting rifle, initial encounter |
|  | W33.03XA | Accidental discharge of machine gun, initial encounter |
|  | W33.09XA | Accidental discharge of other large firearm |
|  | W34.00XA | Accidental discharge from unspecified firearms or gun, initial encounter |
|  | W34.09XA | Accidental discharge from other specified firearms, initial encounter |
|  |  |  |
| Assault | X93.XXXA | Assault by handgun discharge, initial encounter |
|  | X94.0XXA | Assault by shotgun, initial encounter |
|  | X94.1XXA | Assault by hunting rifle, initial encounter |
|  | X94.2XXA | Assault by machine gun |
|  | X94.8XXA | Assault by other larger firearm discharge |
|  | X94.9XXA | Assault by unspecified firearm discharge, initial encounter |
|  | X95.9XXA | Assault by unspecified larger firearm discharge, initial encounter |
|  |  |  |
| Undetermined | Y22.XXXA | Handgun discharge, undetermined intent, initial encounter |
|  | Y23.0XXA | Shotgun discharge, undetermined intent |
|  | Y23.1XXA | Hunting rifle discharge, undetermined intent |
|  | Y23.2XXA | Military firearm discharge, undetermined intent |
|  | Y23.3XXA | Machine gun discharge, undetermined intent |
|  | Y23.8XXA | Other larger firearm discharge, undetermined intent |
|  | Y23.9XXA | Unsepcified larger firearm discharge, undetermined intent |
|  | Y24.9XXA | Unspecified firearm discharge, undetermined intent, initial encounter |
|  |  |  |
| Legal involvement | Y35.001A | Legal intervention involving unspecified firearm discharge, law enforcement official injured |
|  | Y35.002A | Legal intervention involving unspecified firearm discharge, bystander injured |
|  | Y35.003A | Legal intervention involving unspecified firearm discharge, suspect injured |
|  | Y35.009A | Legal intervention involving unspecified firearm discharge, unspecified person injured, initial encounter |
|  | Y35.011A | Legal intervention involving injury by machine gun, law enforcement official injured |
|  | Y35.012A | Legal intervention involving injury by machine gun, bystander injured |
|  | Y35.013A | Legal intervention involving injury by machine gun, suspect injured |
|  | Y35.021A | Legal intervention involving injury by handgun, law enforcement official injured |
|  | Y35.022A | Legal intervention involving injury by handgun, bystander injured |
|  | Y35.023A | Legal intervention involving injury by handgun, suspect injured, initial encounter |
|  | Y35.091A | Legal intervention involving other firearm discharge, law enforcement official injured |
|  | Y35.092A | Legal intervention involving other firearm discharge, bystander injured |
|  | Y35.093A | Legal intervention involving other firearm discharge, suspect injured |
|  |  |  |
|  |  |  |
| Exclude: Self-Harm, Air, BB, pellet, paintball, flare, gun malfunction | | |
